# Pitch perception in school-aged children: Pure tones, resolved and unresolved harmonics

**DOI:** 10.1121/10.0034894

**Published:** 2025-01-29

**Authors:** Jami Fung, Kelly L. Whiteford, Anahita H. Mehta, Bonnie K. Lau

**Affiliations:** 1 Department of Otolaryngology-Head and Neck Surgery, University of Washington, Seattle, Washington 98103, USA; 2 Department of Otolaryngology-Head and Neck Surgery, University of Michigan, Ann Arbor, Michigan 48109, USA jamifht@uw.edu, klwhit@umich.edu, anamehta@umich.edu, blau@uw.edu

## Abstract

Pitch perception affects children's ability to perceive speech, appreciate music, and learn in noisy environments, such as their classrooms. Here, we investigated pitch perception for pure tones as well as resolved and unresolved complex tones with a fundamental frequency of 400 Hz in 8- to 11-year-old children and adults. Pitch perception in children was better for resolved relative to unresolved complex tones, consistent with adults. The younger 8- to 9-year-old children had elevated thresholds across all conditions, while the 10- to 11-year-old children had comparable thresholds to adults.

## 1. Introduction

Children's ability to perceive pitch facilitates listening and learning in noisy environments, like classrooms. Pitch contributes to speech comprehension, music appreciation, and sound recognition. In speech, pitch contours convey emotions (Frick, 1985) and can alter word meanings in tonal languages. Pitch also helps differentiate a target talker from distracting talkers in noisy settings (Qin and Oxenham, 2005). Most environmental sounds, including musical notes and vowels, are harmonic complex tones (HCTs), with frequency components that are integer multiples of a fundamental frequency (*F*0), and their perceived pitch typically corresponds to the *F*0. Low numbered harmonics produce distinct excitation patterns on the basilar membrane (resolved) while higher harmonics, which fall within a single auditory filter, rely on temporal envelope cues for pitch extraction (unresolved, e.g., Glasberg and Moore, 1990; Shackleton and Carlyon, 1994).

Models of pitch perception are generally categorized into three groups: rate-place models, where pitch is encoded by the place of peak stimulation and auditory nerve firing rate (Cohen *et al.*, 1995; Terhardt, 1974; Wightman, 1973); temporal models, where pitch is conveyed by time intervals between phase-locked spikes in the auditory nerve (Cariani and Delgutte, 1996; Licklider, 1951; Meddis and O'Mard, 1997); and spectrotemporal models, which combine tonotopic and phase-locked information (Shamma and Klein, 2000). While extensive data in adults exists for evaluating these models, fewer studies have investigated complex pitch perception during development.

Several studies have investigated children's perception of single frequency pure-tone pitch (Buss *et al.*, 2014; Halliday *et al.*, 2008; Jensen and Neff, 1993; Maxon and Hochberg, 1982; Thompson *et al.*, 1999), pitch perception for HCTs (Buss *et al.*, 2017; Deroche *et al.*, 2012; Deroche *et al.*, 2014; Rocheron *et al.*, 2002), dynamic *F*0 pitch sweeps (Deroche *et al.*, 2016), *F*0 sweep direction labeling (Deroche *et al.*, 2019), and voice *F*0 (Nagels *et al.*, 2020, 2024). These studies generally indicate that pitch discrimination in all these cases improves throughout school-age years.

Previous studies indicate that infants can discriminate large *F*0 changes in unresolved harmonics (Lau and Werner, 2014), but the development of *F*0 sensitivity to unresolved harmonics is unknown (Lau *et al.*, 2021; Lau and Werner, 2012). In children ages 5–11, pitch discrimination thresholds for the syllable /ba/ with a 250 Hz *F*0 were similar to 250 Hz pure tone thresholds, reaching adult-like thresholds at around 11.5 years (Buss *et al.*, 2017). Deroche *et al.* (2012) found that *F*0 discrimination thresholds for a 100 Hz broadband sine-phase HCT, which contained both spectral and temporal cues for pitch, were about nine times better than amplitude modulation (AM) rate discrimination thresholds for a 100 Hz sinusoidally amplitude‐modulated noise, which relies on temporal envelope cues for pitch, with no age effect observed in children ages 6–15. However, it is unclear in the aforementioned studies whether the children were truly basing their judgements on the *F*0 pitch or using individual components to make their judgements.

In this study, we measured pitch discrimination thresholds for HCTs with resolved and unresolved harmonics and compared them to thresholds for pure tones with the same *F*0. To ensure that perceptual judgements were based on the *F*0 pitch, the lowest harmonic was roved (Houtsma and Smurzynski, 1990; Mehta and Oxenham, 2020; Micheyl *et al.*, 2010). Our study aimed to address two questions: (1) What is the effect of age on pitch discrimination for pure tones, resolved, and unresolved harmonics? (2) How do pitch discrimination thresholds differ among these three conditions?

## 2. Methods

### 2.1 Participants

**Fig. 1.**
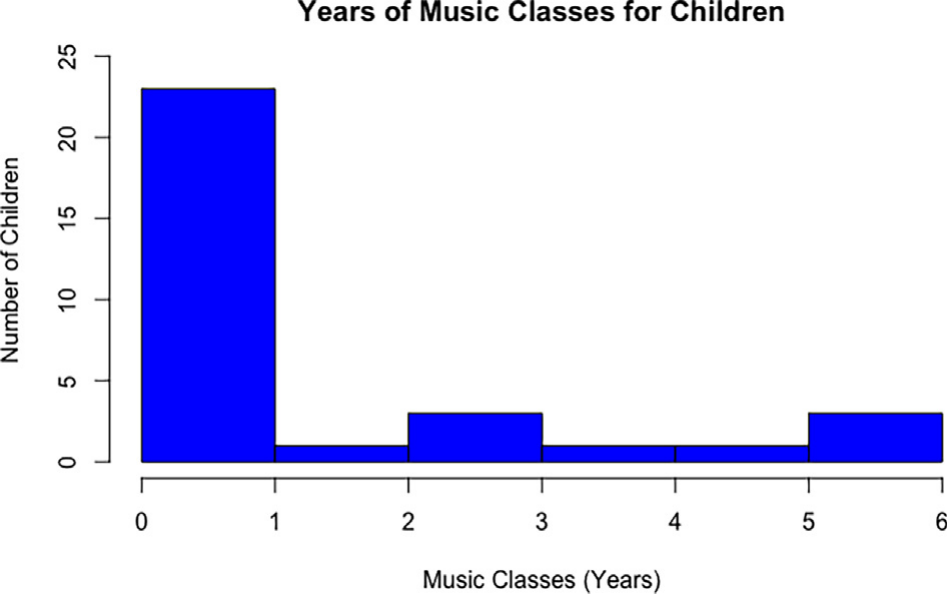
Years of music classes for children.

Thirty-two children (ages 8–11) and 16 adults (ages 18–32) were tested. The children were divided into two groups: younger (8–9 years; ten males, six females) and older (10–11 years; three males, 13 females). The adults included four males and 12 females. Equal sample sizes were maintained across the three age groups. All participants were recruited from the University of Washington (UW) Communication Studies Participant Pool, which is a database of infants, children, and adults interested in participating in research and a post on the UW Institute for Translational Health Sciences Participate in Research website. Inclusion criteria were self-reported typical hearing and geometric mean pure-tone pitch discrimination thresholds < 20%. The latter criterion was adopted as a means of assessing task compliance and to ensure listeners could discriminate *F*0 with a reasonable degree of accuracy. All but one participant spoke English as their primary language, based on daily hours of usage. Nine adults and three children spoke a second language. All adults had five or fewer years of formal music classes and all children had six or fewer years (Fig. 1), with most children having no formal musical training. For children, years of music classes was not related to performance in any pitch condition [*p* > 0.05 in pure tone (PT), low number resolved harmonics (RES), and high number unresolved harmonics (UNRES)].

An additional 11 children and two adults were excluded. Nine children (mean age = 10.77, range = 8–16 years, one female, mean years of music classes = 0.75, range = 0–3 years) exceeded the pure-tone pitch discrimination cutoff. Two children (both age 9, one female) were excluded due to technical difficulties with slow internet speed. One 21-year-old female was unable to complete the study, and another 22-year-old female was excluded due to background noise. Adult participants and child caregivers provided consent remotely via research electronic data capture (REDCap), and all participants were compensated with an electronic gift card. All procedures and protocols were approved by the University of Washington Institutional Review Board.

### 2.2 Stimuli

Discrimination thresholds were measured for three conditions: PT, RES, and UNRES. The *F*0 of all stimuli was 400 Hz to facilitate audibility of tones presented on a home-based computer.

In all conditions, three tones were presented per trial: one target and two reference tones. The target *F*0 was always higher than the reference *F*0. All stimuli were 500 ms in duration with 10 ms raised cosine onset and offset ramps, separated by a 50 ms inter-stimulus interval. Complex tones were presented in threshold equalizing noise (TEN) (Moore *et al.*, 2000) ranging from 20 Hz to 20 kHz to mask distortion products and to ensure a similar sensation level of all frequency components, whereas PT stimuli were presented in quiet. RES and UNRES stimuli were comprised of six consecutive harmonics presented at full amplitude, spectrally flanked by two harmonics presented 6 dB lower, for eight consecutive harmonics in total. The level of edge components was attenuated to reduce salience of potential edge-pitch cues (Kohlrausch and Houtsma, 1992). The nominal lowest harmonic number of the full-amplitude components, *N*, was 3 and 13 in the RES and UNRES conditions, respectively, and roved across trials. Thus, the actual lowest harmonic number would be either *N* or *N* + 1, after being selected randomly without replacement in each trial. The components in each HCT were added in sine phase. The TEN was presented 10 dB lower than the per component level of the complex tones (before level roving), with the estimated equivalent rectangular bandwidth (ERB_N_) of the auditory filter being 1 kHz. In each trial, a lowpass filter with a cutoff frequency half an octave lower than the nominal frequency of the lowest harmonic was applied to an additional (independent) TEN, which was added at a level per ERB_N_ equivalent to the level per full amplitude component. The TEN had the same 10 ms raised cosine ramps and was gated on 200 ms before the onset of the first tone and was gated off 100 ms after the offset of the third tone.

### 2.3 Procedure

Demographic, language, and musical experience information were collected via an online questionnaire on REDCap before the video session. Participants then joined the experimenter's Zoom meeting room using a laptop or desktop with a webcam. The psychoacoustic task was administered remotely via matlab Webapps (Mathworks, Natick, MA) during the online session and completed at the participants' home or other quiet locations. Before the experiment, participants were instructed to turn on their webcam and share their computer screen and sound to allow the experimenter to monitor their behavior and troubleshoot any technical issues. Participants were asked to ensure a quiet environment free from distractions, gadgets, pets, and family members. Participants listened to the sound stimuli through headphones. Prior to the experiment, a sound check was conducted to confirm comfortable sound levels. The experimenter was muted during the task to minimize noise.

Thresholds were obtained using a three-interval, three-alternative forced-choice task with an adaptive two-down-one-up procedure to estimate the 70.7% correct point of the psychometric function (Levitt, 1971). On each trial, the target tone was presented in one of the three intervals, selected at random. The three intervals were displayed visually on the participant's computer screen as rectangular boxes labeled with the numbers “1,” “2,” and “3,” which illuminated red simultaneously with the onset of each tone. The participants were instructed to select the interval containing the different tone by clicking the corresponding box on the screen. The same instructions were provided to both adult and child participants, and no practice was provided. After three trials on the first run, the experimenter asked the participants to pause and confirmed audibility of stimuli and task comprehension.

The three tones presented had fundamental frequencies (*F*0s) that were geometrically centered around the nominal *F*0. The initial *F*0 difference (*ΔF0*) between the two tones was 20%, relative to the lower of the two *F*0s. After one incorrect or two consecutive correct responses, *ΔF0* was increased or decreased by a factor of 1.41, respectively. The step size was reduced to a factor of 1.2 and the run continued for another six reversals after the first four reversals in the adaptive procedure. There were three runs per condition (i.e., PT, RES, and UNRES). The order of conditions was selected at random for each participant and each repetition, so that participants tested all conditions once before they were repeated. The maximum *F*0 difference allowed was 398% relative to the reference *F*0. After six hits of the maximum level, the run would be discarded. This occurred for one adult participant in the UNRES condition. For that participant, we took the average of two runs instead of three. The threshold of each run was derived from the log-transformed data by taking the geometric mean of ΔF0 at the last six reversals. At the end of each run, participants were prompted to press “s” on their keyboard to start a new run or “e” to end. The experimenter provided periodic reinforcement and rest breaks as need. All participants were asked verbally if they would like to take a break at least once throughout the session. Children with difficulty completing the full test session due to fatigue would have been excluded from the analysis based on their high pure tone thresholds. The entire experiment session took approximately 40–50 min.

## 3. Results

**Fig. 2.**
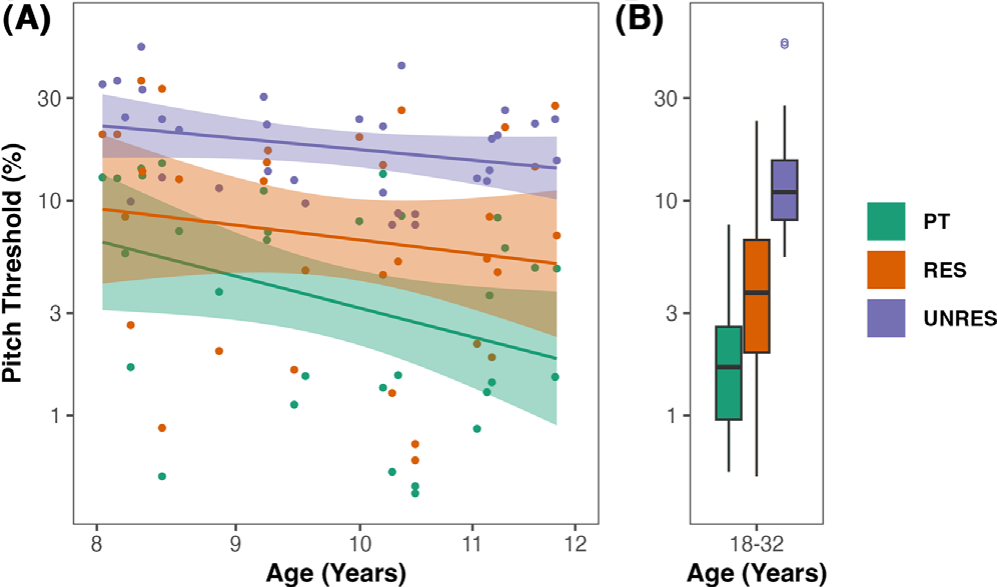
Individual pitch discrimination thresholds as a function of age in the PT, RES, and UNRES conditions for children. Dots indicate individual child participants in (A) and adult participants in (B). The solid lines are the regression lines of the pitch thresholds on age and 95% confidence intervals are indicated by the shaded area.

**Fig. 3.**
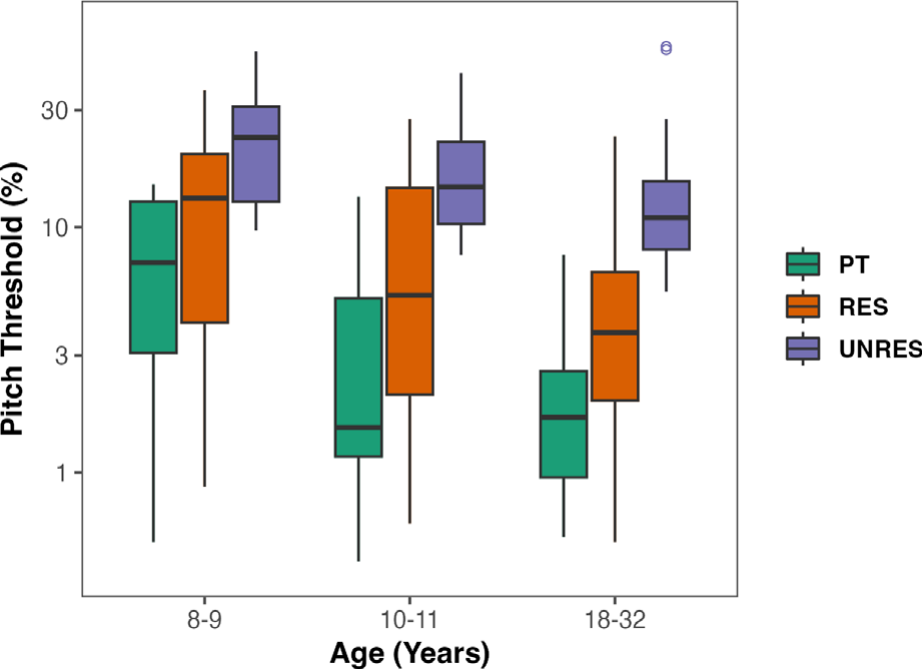
Pitch discrimination thresholds as a function of age group in the PT (green), RES (red), and UNRES (blue) conditions.

The mean and standard deviation of thresholds across the conditions and age groups are shown in Table 1. All statistical analyses were conducted on log-transformed data to ensure that the variance was roughly equal across conditions. A repeated measures analysis of variance (rmANOVA) was performed on the pitch discrimination thresholds, with condition (PT, RES, UNRES) as the within-subjects factor, and age group (adult, older children, younger children) as the between-subjects factor. Degrees of freedom were adjusted by a Huynh–Feldt epsilon value of 0.86 when the assumption of sphericity was violated. Significant effects were interpreted using *post hoc t* tests with Bonferroni correction. Results revealed significant main effects of age group (*F*_2, 45_ = 4.05, *p* = 0.024, η^2^_g_ = 0.12) and condition (*F*_1.73, 77.70_ = 142.92, *p* < 0.001, η^2^_g_ = 0.40) but no significant interaction (*F*_3.45, 77.70_ = 1.94, *p* = 0.122, η^2^_g_ = 0.02). Pitch discrimination was lowest (best) for pure tones, followed by resolved harmonics and finally unresolved harmonics, with unresolved thresholds being the poorest of the three (*p* < 0.001 for all contrasts) (Figs. 2 and 3). Pitch discrimination thresholds also decreased with participant age, with thresholds being the highest for the youngest participant group. Pitch discrimination thresholds were significantly different between the younger and older children (*p* = 0.002), and between the younger children and adults (*p* = 0.0002) but not between older children and adults (*p* = 0.633).

**Table 1.** Descriptive statistics of pitch thresholds.

	Condition					
	PT		RES		UNRES	
Age group (years)	*M* ^a^	*SD* ^b^	*M*	*SD*	*M*	*SD*
8– 9	7.63	5.01	13.81	10.66	23.29	11.83
10–11	3.68	3.75	9.17	9.11	17.22	9.18
18–32	2.34	2.03	6.23	6.59	16.78	15.43
All participants	4.55	4.35	9.74	9.30	19.10	12.51

^a^ Mean (M).

^b^ Standard deviation (SD).

## 4. Discussion

This study was the first to investigate the discrimination of unresolved harmonics in children between 8–11 years of age and compared performance across pure tone, resolved, and unresolved harmonic conditions at the same *F*0. Importantly, necessary stimulus controls, including the masking of distortion products and roving of lowest harmonic number, were employed to ensure that participants were discriminating on the basis of *F*0, as opposed to other spectral cues. An additional novel result of this study is that reliable pitch discrimination thresholds can be obtained remotely from children as young as 8 years of age. We observed a significant main effect of age on pitch discrimination thresholds. Children in the younger age group performed worse than adults, but by 10–11 years of age, children performed comparably to adults across conditions. The effect of condition was also found to be significant, with the best thresholds observed in the PT condition and the worst thresholds in the UNRES condition.

These results are in line with earlier studies in which adult PT thresholds were reported to range roughly from 0.1%–20% (Buss *et al.*, 2017; Moore, 1973; Whiteford and Oxenham, 2017). Children also showed performance comparable to previous findings reported by Buss *et al.* (2017) at 250 Hz, as PT threshold improvements were observed with increasing age, reaching the adult mean of ∼2% by 10–11 years. While significant variability was observed in each age group, the variability was comparable to past studies, even though we tested participants remotely.

While mean PT thresholds were comparable to past studies, mean RES thresholds were higher than previously reported. Deroche *et al.* (2012) measured thresholds of ∼1% for a 100 Hz *F*0 for children 6–16 years of age, while the mean RES thresholds for both younger and older children in this present study were much higher. The most likely reason for these threshold elevations across all age groups is due to roving the lowest harmonic present in the HCT; this creates uninformative changes in timbre that may be distracting and elevate thresholds (Allen and Oxenham, 2014; Lau *et al.*, 2021). For example, Allen and Oxenham (2014) measured *F*0 discrimination thresholds with changes in timbre in adults and found mean thresholds around 3%–4% for non-musicians; all participants in the present study had limited musical training (<6 years). It is important to note however, that the best performing children and adults in this study did perform as well as the best performing children in Deroche *et al.* (2012) and the best performing adults in Mehta and Oxenham (2020), with thresholds of ∼0.5%. Furthermore, we tested an *F*0 of 400 Hz as opposed to 100 Hz, and while thresholds at these two *F*0s should be comparable for adults (Mehta and Oxenham, 2020), whether this is the case for children is unknown. Past studies of PT frequency discrimination conducted in infants and children show that by 6 months of age, frequency discrimination at 4000 Hz is approaching adult levels (Olsho, 1984; Olsho *et al.*, 1987). However, low frequency thresholds at ∼500 Hz are only slightly better than infants at 4–6 years and gradually improve through childhood (Hill *et al.*, 2005; Jensen and Neff, 1993; Maxon and Hochberg, 1982).

These findings are also consistent with previous studies reporting that thresholds of unresolved harmonics are worse than those of the resolved harmonics (Houtsma and Smurzynski, 1990; Bernstein and Oxenham, 2003). This suggests that unresolved harmonics produce a less salient pitch for children as they do for adults. It is possible that additional cognitive or non-sensory factors may contribute to this challenge for children; however, we do not see an interaction between age and condition suggesting that these non-sensory influences do not affect children in the UNRES condition more than RES or PT.

Our finding that pitch thresholds across conditions are adult-like by 10–11 years of age is inconsistent with past studies of 100 and 200 Hz *F*0 discrimination (Deroche *et al.*, 2014), swept *F*0 discrimination (Deroche *et al.*, 2016), swept *F*0 direction labelling (Deroche *et al.*, 2019), and voice *F*0 discrimination (Nagels *et al.*, 2020), which showed continued improvement in performance beyond 10 years of age and well into adolescence. One possible difference for the seemingly discrepant age of maturation is the different study design and sample size. In this present study, 32 participants were recruited across two small age bins, resulting in 16 participants per age interval. However, in the other studies, age was treated as a continuous variable and participants were recruited across a large age range, resulting in fewer participants of each age. Due to the variability in pitch perception observed across individuals (including both children and adults), larger sample sizes are required per age group. Of course, the stimuli employed across these studies also differ significantly and dynamic swept *F*0 sensitivity and direction labeling are also very different tasks.

Adult thresholds in the two harmonic complex conditions, ∼6% in RES and ∼16% in UNRES, were higher than those reported inWe ha Mehta and Oxenham (2020), where a threshold of ∼8% was observed for unresolved harmonics and a threshold of ∼1% was observed for resolved harmonics. One possible reason for this discrepancy in adult UNRES thresholds is that we adopted a comparatively lax PT pitch threshold inclusion criterion, <20% at 400 Hz, while Mehta and Oxenham (2020) had an inclusion criterion of <2% *F*0 threshold at 200 Hz. Additionally, since testing took place remotely, it is possible that some at-home speakers were unable to produce the frequencies of our highest harmonics (∼8 kHz) for the unresolved condition. Nonetheless, as in the PT and RES conditions, our best performing participants, both children and adults, performed as well as the best performing participants in Mehta and Oxenham (2020). Thus, it may be that we see increased variability overall with remote testing, resulting in elevated mean thresholds. For child thresholds in the UNRES condition, no comparisons can be made as no prior studies have been conducted. However, Lau and Werner (2014) showed that infants were able to categorize missing fundamental complexes composed of unresolved harmonics by 3 months of age.

Although data were collected remotely using participants' personal devices, the best performing participants across all age groups had thresholds consistent with past literature for all conditions. Even some of the youngest participants (8 to 9 years of age) had thresholds of <1% for PT and RES conditions. One likely reason for these good thresholds is the presence of an online experimenter throughout the whole experiment, who provided behavioral support and monitored participant compliance and engagement. This ensured functioning audio and enabled better performance on the task.

In summary, this study found that by age 10–11 years, children had comparable pitch perception to adult listeners for pure tones as well as resolved and unresolved harmonics. Children 8–9 years of age had elevated thresholds across conditions. One possibility is that pitch processing is not adult-like until 10–11 years of age. Another possibility is the influence of non-sensory factors, such as attention and working memory, contributed to poorer discrimination. Overall, children had the poorest thresholds in the unresolved condition, demonstrating that temporal-envelope pitch is less salient for children as it is for adults. Regarding models of pitch perception, these findings suggest that children between 8 and 11 years of age have access to both spectral and temporal envelope cues for pitch, although the design of the study does not allow us to differentiate which cues were used in the PT and RES conditions. Future studies comparing remote vs in-laboratory data collection are warranted with a larger sample of participants, in addition to more experiments across a wider range of *F*0s to characterize the effect of harmonic resolvability in development. While these results suggest that by 10–11 years of age, children show comparable pure tone, resolved, and unresolved harmonic discrimination at 400 Hz to adults, whether they can use these pitch cues for speech perception or noisy, real-world auditory discrimination requires further investigation.

## Data Availability

The datasets generated during and/or analyzed during the current study are available from the corresponding author on reasonable request.
